# The Efficacy of Combining Antiangiogenic Agents with Chemotherapy for Patients with Advanced Non-Small Cell Lung Cancer Who Failed First-Line Chemotherapy: A Systematic Review and Meta-Analysis

**DOI:** 10.1371/journal.pone.0127306

**Published:** 2015-06-02

**Authors:** Jin Sheng, Yunpeng Yang, Yuxiang Ma, Bijun Yang, Yaxiong Zhang, Shiyang Kang, Ting Zhou, Shaodong Hong, Tao Qin, Zhihuang Hu, Wenfeng Fang, Yan Huang, Li Zhang

**Affiliations:** 1 Department of Medical Oncology of Sun Yat-sen University Cancer Center, Guangzhou, China; 2 State Key Laboratory of Oncology in South China, Guangzhou, China; 3 Collaborative Innovation Center for Cancer Medicine, Guangzhou, China; 4 Zhongshan School of Medicine, Sun Yat-sen University, Guangzhou, China; Catalan Institute of Oncology, SPAIN

## Abstract

**Background:**

The clinical outcomes of patients with NSCLC who progressed after first-line treatments remain poor. The purpose of this study was to assess the advantage of antiangiogenic therapy plus standard treatment versus standard treatment alone for this population of patients.

**Methods:**

We conducted a rigorous search using electronic databases for eligible studies reporting antiangiogenic therapy combined with standard second-line chemotherapy versus standard second-line treatment for patient who progressed after front-line treatment. Pooled risk ratio and 95% confidence intervals were calculated using proper statistical method. Predefined subgroup analyses were conducted to identify the potential proper patients.

**Results:**

Thirteen phase II/III RCTs which involved a total of 8358 participants were included. Overall, there was significant improvement in OS (HR 0.94, 95%CI: 0.89-0.99, p=0.03), PFS (HR 0.80, 95%CI: 0.76-0.84, p<0.00001), ORR (RR 1.75, 95%CI: 1.55-1.98, p<0.00001) and DCR (RR 1.23, 95%CI: 1.18-1.28, p<0.00001) in the group with antiangiogenic therapy plus standard treatment versus the group with standard treatment alone. Subgroup analysis showed that OS benefit was presented only in patients treated with docetaxel plus antiangiogenic agents (HR 0.92, 95%CI: 0.86-0.99, p=0.02) and patients with non-squamous NSCLC (HR for OS 0.92, 95%CI: 0.86-0.99, p=0.02).

**Conclusions:**

This study revealed that the addition of antiangiogenic agents to the standard treatments could provide clinical benefit to NSCLC patients who failed their first-line therapy. Furthermore, proper selection of the combined standard cytotoxic agent, as well as the patient population by tumor histology, is warranted for future studies and clinical application of antiangiogenic therapy.

## Introduction

Although several targeted therapies against driver mutations have been recently developed and led to extraordinary clinical benefit for NSCLC patients, more than half of the patients without known driver mutations still lack chance for targeted therapies [1]. The first-line treatment for these patients typically includes four to six cycles of platinum-based chemotherapy, and about 70% of patients could achieve clinical remission or disease stabilization [2, 3]. However, almost all patients would experience disease progression and eventually need subsequent therapies.

Currently the recommended second-line or third-line treatments for NSCLC patients include single-agent docetaxel, erlotinib, pemetrexed or gemcitabine [2, 4–6]. Clinical outcomes in this population continue to be poor, with an overall survival (OS) of 7 to 9 months, progression-free survival (PFS) of 2 to 4 months, and objective response rate (ORR) of less than 10% [7]. Therefore, novel treatment strategies for advanced NSCLC patients failing the first-line therapies are urgently required.

Angiogenesis plays an important role in cancer development. Several agents with antiangiogenic effect have been developed, including small-molecule multiple receptor tyrosine kinase inhibitors (TKIs, such as sunitinib, vandetanib, nintedanib and sorafenib), and monoclonal antibodies (MAs, such as bevacizumab, ramucirumab, and aflibercep). Previous studies were conducted to test the hypothesis that combining standard therapies and antiangiogenic agents might confer additional clinical benefit in advanced NSCLC patients. Eastern Cooperative Oncology Group 4599 study demonstrated that the addition of antiangiogenic agent (bevacizumab) to the standard chemotherapy could improve OS of NSCLC patients treated in the first-line setting [8]. Additionally, more than 10 studies evaluated the effectiveness of the combination therapy strategy in patients who failed their first-line treatment. However, the outcome results of these studies were inconsistent.

The role of antiangiogenic therapy has been well recognized in first-line treatment for NSCLC patients. Two meta-analysis indicated significant improvement of ORR, PFS, and OS for the combination of antiangiogenic agent (bevacizumab) and chemotherapy compared with chemotherapy alone [9, 10]. Several clinical guidelines also recommend the addition of bevacizumab to the standard treatment in the first-line setting [11, 12]. However, the advantage of adding antiangiogenic agent to the standard treatment in patients who failed from first-line therapy is still confusing. Therefore, this meta-analysis was performed to compare the efficacy of angiogenesis inhibitors plus standard treatment versus standard treatment alone for patients with advanced NSCLC that progressed after first-line treatment. Predefined subgroup analysis were conducted to identify the potential proper patients.

## Methods

### Search strategy

In October 2014, all relevant articles were retrieved by searching through PubMed, Embase and the Central Registry of Controlled Trials of the Cochrane Library, as well as the ASCO and ESMO databases. Search strategety were the combination of “non-small-cell lung cancer” with any of the following: ‘‘angiogenesis inhibitors” or ‘‘sorafenib”, “sunitinib”, ‘‘bevacizumab”, ‘‘vandetanib”, ‘‘aflibercept”, ‘‘nintedanib”, ‘‘pazopanib”,”ramcirumab” or ‘‘axitinib”. Recent reviews and references of the included studies and were checked manually as a supplement. No language restriction was applied.

### Eligibility criteria

Studies that met the following criteria were included: (1) Adult (≥18 years) patients with histologically or cytologically confirmed stage IIIB/IV NSCLC (all histologies); (2) Phase II or III RCTs that evaluate the efficacy of angiogenesis inhibitors plus a present standard single agent chemotherapy (pemetrexed, doctaxel or erlotinib) as salvage cure for patients progressing after first-line treatment; (3) The control group must be the corresponding cytotoxic agent; (4) At least one endpoints (PFS, OS, ORR and DCR) was reported. Trials were excluded if they fail to meet the including criteria. In cases of duplicate trials, the most complete reports were included.

### Definition of angiogenesis inhibitors

Angiogenesis inhibitors were defined as agent blocking angiogenic pathways mediated by vascular endothelial growth factor receptor (VEGFR). Oral small-molecule TKIs or monoclonal antibodies were classified as two types of angiogenesis inhibitors.

### Quality assessment and data extraction

The data collection and assessment of methodological quality followed the QUORUM and the Cochrane Collaboration guidelines (http://www.cochrane.de). Researcher evaluated the quality of each eligible study according to the JADAD score [13].

Baseline clinical characteristics, total number of enrolled participants, the risk ratio (RR) and 95% confidence intervals (CI) for objective response rates (ORR) and disease control rates (DCR), median value, hazard ratio (HR) of overall survival (OS) and progression-free survival (PFS), were extracted by two investigators independently. Discrepancies were discussed by the third investigators to reach consensus. We tried to obtain additional unpublished data by contacting the primary authors. Meta-analyses was conducted according to the Preferred Reporting Items for Systematic Reviews and Meta-Analyses (PRISMA) statements as shown in S1 Checklist.

The primary outcome was set as OS. Second outcomes included PFS, ORR and DCR. The extracted data of OS, PFS, ORR and DCR were pooled. Further exploration was conducted by subgroup analysis of survival outcomes according to the histological type (selective population for squamous carcinoma and non-squamous carcinoma), the second-line chemotherapy agents (pemetrexed, doctaxel, erlotinib) and the classification of angiogenesis inhibitors (TKI or monoclonal antibody).

### Statistical analysis

We defined the experimental arm as angiogenesis inhibitors-containing group. The control arm was standard second-line single agent chemotherapy. Heterogeneity across studies was assessed with a forest plot and the inconsistency statistic (I^2^). A random-effects model was employed in case of the existence of potential heterogeneity (I^2^≥50%); otherwise, the fixed-effect model was applied. Pooled hazard ratios (HRs) for survival outcomes (PFS and OS) and pooled risk ratio (RRs) for dichotomous data (ORR, DCR) with 95% CI were calculated using the proper algorithm. All calculations and assessment of the risk of bias were performed by Review Manager (version 5.2 for Windows; the Cochrane Collaboration, Oxford, UK). Graphical funnel plots were generated to visually inspect for publication bias. P<0.05 was considered statistically significant for all analysis.

## Results

### Study characteristics

**Fig 1:**
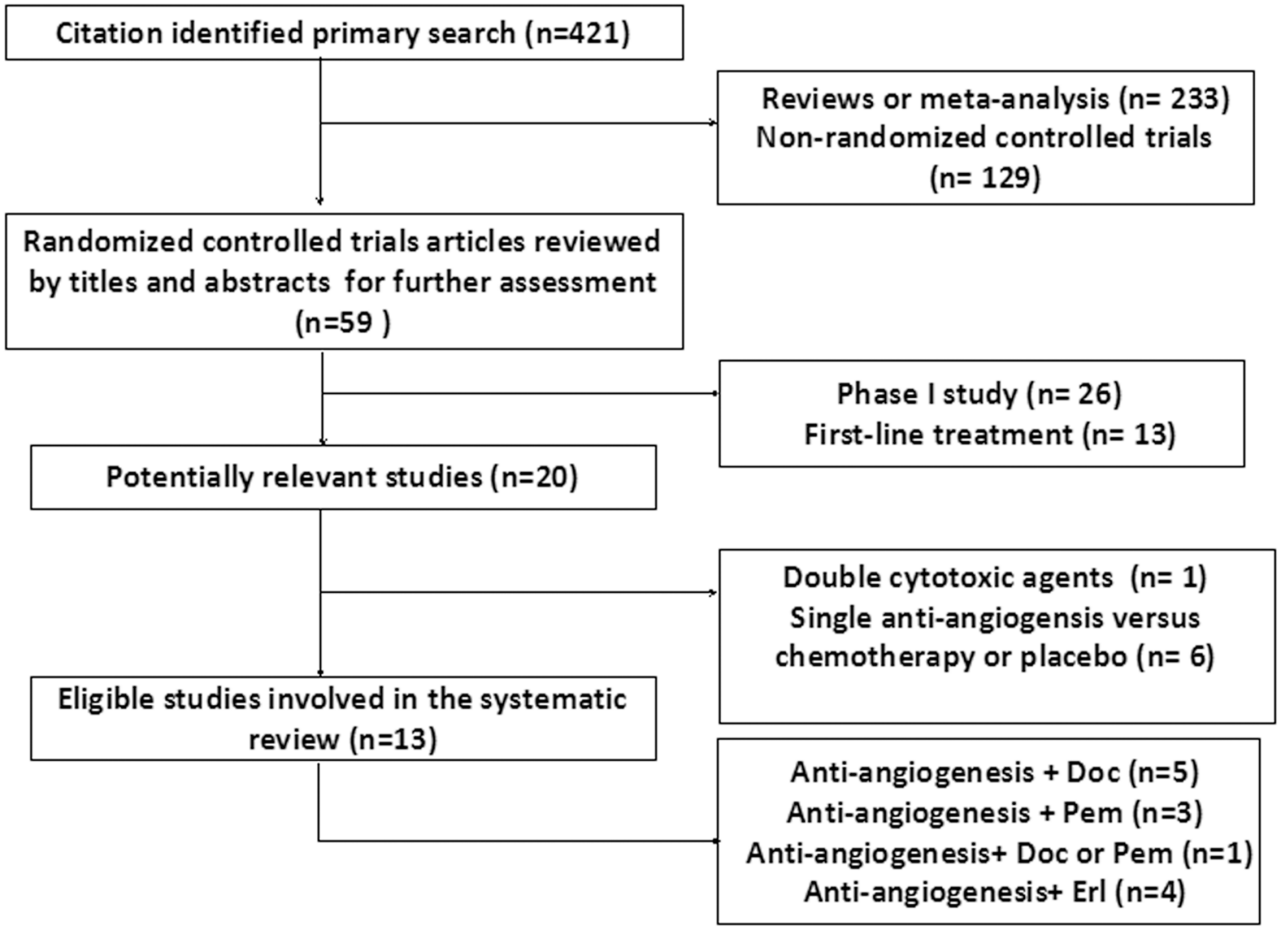
The flowchart of the process for selecting relevant articles.

Twenty potentially eligible trials were rigorously identified by full-text review, 7 of which were excluded for reasons listed in Fig 1. Finally, 13 studies with 8358 patients met the inclusion criteria and were included for the analysis. In respect to the type of standard second-line cytotoxic agents, the number of studies involving pemetrexed, docetaxel and EGFR-TKI were 3 [14–16], 5 [17–21], 4 [22–25], respectively. Another one [26] was designed to illustrate the efficacy of the addition of bevacizumab to docetaxel or pemetrexed. Four studies [19, 20, 22, 26] reported the result of combination of antiangiogenic monoclonal antibodies, and the remaining nine studies were about single agent chemotherapy combined with VEGF-TKI or placebo. In further subgroup exploration, the efficacy of “double TKIs” model, which implies antiangiogenic TKI combined with EGFR-TKI, was evaluated based on 3 RCTs [23–25]. Regarding histological type, nine studies provided relevant subgroup information. The specific number of the studies included may vary according to the corresponding outcomes. Detailed information of included studies and the result of quality assessment were listed in Table 1. Apparently, all of the trials were qualified enough to be included as the Jadad Score are all at least 3.

**Table 1:** Characteristics of included studies and agents.

Author	Year	phase	line	Arms	No. of enrolled patients	Percent of non-squamous cancer (%)	Median PFS (months)	Median OS (months)	ORR(event)	DCR(event)	Jadad score
de Boer	2011	III	2	Vandetanib + Pem	256	79	4.1	10.5	49	146	4
				Placebo + Pem	278	78	2.8	9.2	22	128	4
Hanna	2013	II	2	Nintedanib + Pem	353	100	4.4	12.2	33	215	4
				Placebo + Pem	360	100	3.6	12.7	30	192	4
Heist	2014	II	≥2	Sunitinib + Pem	41	85	3.7	6.7	9	30	3
				Placebo + Pem	42	90	4.9	10.5	6	27	3
Heymach	2007	II	2	Vandetanib + Doc	42	88	18.7	13.1	11	35	3
				Placebo + Doc	41	89	12.0	13.4	5	23	3
Herbst	2010	III	≥2	Vandetanib + Doc	694	73	4.0	10.6	117	434	5
				Placebo + Doc	697	77	3.2	10.0	69	400	5
Ramlau	2012	III	≥2	Aflibercept + Doc	456	100	4.1	10.4	94	277	5
				Placebo + Doc	457	100	5.2	10.1	36	191	5
Reck	2014	III	2	Nintedanib+ Doc	655	57.9	3.5	10.1	29	361	5
				Placebo + Doc	659	57.7	2.7	9.1	22	278	5
Garon	2014	III	2	Ramucirumab+ Doc	628	75	4.5	10.5	145	403	5
				Placebo + Doc	625	73	3.0	9.1	85	329	5
Herbst	2007	II	2	Bevacizumab + Pem/Doc	40	100	4.8	12.6	5	21	3
				Placebo + Pem/Doc	41	100	3.0	8.6	5	16	3
Spigel	2011	II	≥2	Sorafenib + Erl	112	70	3.4	7.6	40	60	4
				Placebo + Erl	56	69	1.9	7.2	12	21	4
Herbst	2011	III	2	Bevacizumab + Erl	319	97	3.4	9.3	117	434	5
				Placebo + Erl	317	95	1.7	9.2	69	400	5
Scagliotti	2012a	III	≥2	Sunitinib + Erl	480	71.9	3.6	8.2	52	209	5
				Placebo + Erl	480	71.9	2.0	7.6	34	170	5
Groen	2013	II	≥2	Sunitinib + Erl	65	77	2.8	9.0	3	NA	5
				Placebo + Erl	67	72	2.0	8.5	2	NA	5

Note: Pem for pemetrexed; Doc for doctaxel; Erl for erlotinib; PFS means progression-free survival ans OS means overall survival; ORR means objective response rate; DCR means disease control rate.

### Primary outcome: OS

**Fig 2:**
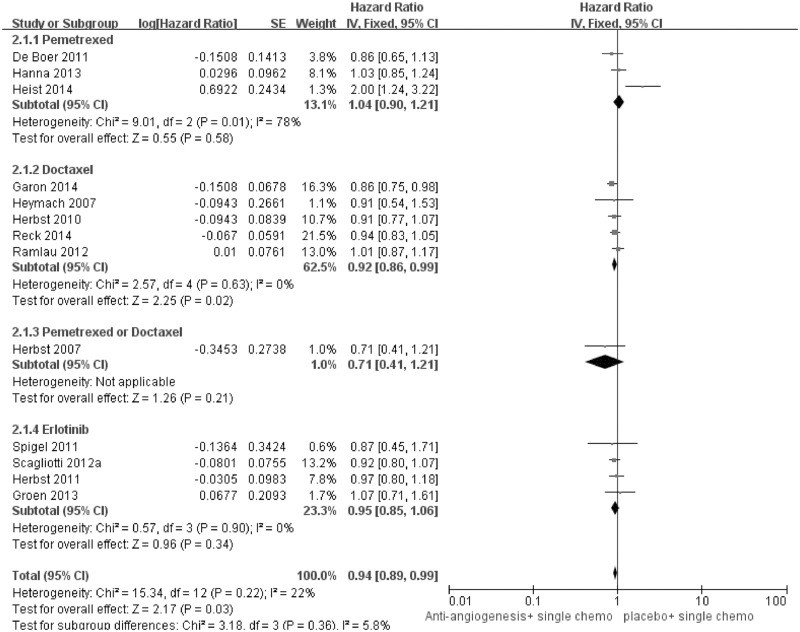
Forest plot and pooled HR & 95%CI for OS: Antiangiogenic agents plus single agent chemotherapy versus standard second-line chemotherapy.

13 studies met the inclusion criteria and were finally included for OS analysis (Fig 2). In general, for patients who progressed after front-line chemotherapy, the addition of angiogenesis inhibitors was associated with modest but significant survival improvement compared with standard second-line single cytotoxic agent, reducing 6% of the risk of death (HR for OS 0.94, 95%CI: 0.89–0.99, p = 0.03). (Table 2)

**Table 2:** Summary of the pooled results and corresponding details.

	No. of articles	Pooled HR or RR with 95%CI	P-value	Heterogeneity (I^2^)	Analysis model
**OS**	13	0.94 (0.89–0.99)	0.03	22%	Fixed
**PFS**	13	0.80 (0.76–0.84)	<0.00001	31%	Fixed
**ORR**	13	1.75 (1.55–1.98)	<0.00001	12%	Fixed
**DCR**	12	1.23 (1.18–1.28)	<0.00001	43%	Fixed

Although one study [14] presented apparent heterogeneity among the included trials. After the removal of relevant data, the pooled HR for OS was 0.93 (95%CI: 0.88–0.99, p = 0.01). Besides, other individual study was also proved no substantially influence on the overall result.

As listed in Table 3, the pooled result indicated that the patients with non-squamous cancer benefited most from the combination strategy (Pooled HR for OS 0.92, 95%CI: 0.86–0.99, p = 0.02).

**Table 3:** Summary of the subgroup results: Pooled HR & 95%CI for OS.

	No. of articles	Pooled HR with 95%CI	P-value	Heterogeneity (I^2^)	Analysis model
**AT** *	9	0.95 (0.89–1.02)	0.16	30%	Fixed
**AA** ^&^	4	0.93 (0.85–1.01)	0.08	18%	Fixed
**Pemetrexed**	3	1.14 (0.80–1.64)	0.47	78%	Random
**Doctaxel**	5	0.92 (0.86–0.99)	0.02	0%	Fixed
**Non-Doctaxel**	7	0.98 (0.90–1.07)	0.66	43%	Fixed
**EGFR-TKI**	4	0.95 (0.85–1.06)	0.34	0%	Fixed
**Chemotherapy**	9	0.94 (0.88–1.00)	0.05	46%	Fixed
**Double TKI** ^¶^	3	0.94 (0.82–1.07)	0.34	0%	Fixed
**Non-Squamous cancer**	9	0.92 (0.86–0.99)	0.02	10%	Fixed
**Squamous cancer**	6	0.96 (0.87–1.07)	0.50	0%	Fixed
**Non-squamous cancer+AT**	5	0.91 (0.83–1.00)	0.05	0%	Fixed
**Adenocarcinoma**	4	0.90 (0.81–1.00)	0.06	9%	Fixed

* AT for antiangiogenic-TKI;

^&^ AA refers to antiangiogenic antibody;

^¶^ Double TKI means antiangiogenic-TKI plus EGFR-TKI.

In addition, the pooled result was in favor of the combination of docetaxel with angiogenesis, which significantly improved the overall survival for patients progressing after first-line chemotherapy (pooled HR for OS was 0.92, 95%CI: 0.86–0.99, p = 0.02).

Angiogenensis inhibitor combined with pemetrexed or erlotinib slightly improved OS, however, the difference was not significant compared with chemotherapy alone.

With respect to angiogenesis inhibitors, monoclonal antibody was only numerically superior to VEGF-TKI in decreasing the risk of death (Pooled HR were separately 0.93, 95%CI: 0.85–1.01, p = 0.08 and 0.95, 95%CI: 0.89–1.02, p = 0.16).

Meanwhile, the distinguished combination of antiangiogenetic TKI and EGFR-TKI slightly decreased the risk of death, however, the difference was not statistically significant (Pooled HR 0.94, 95%CI: 0.82–1.07, p = 0.34).

### Secondary outcomes: PFS, ORR and DCR

**Fig 3:**
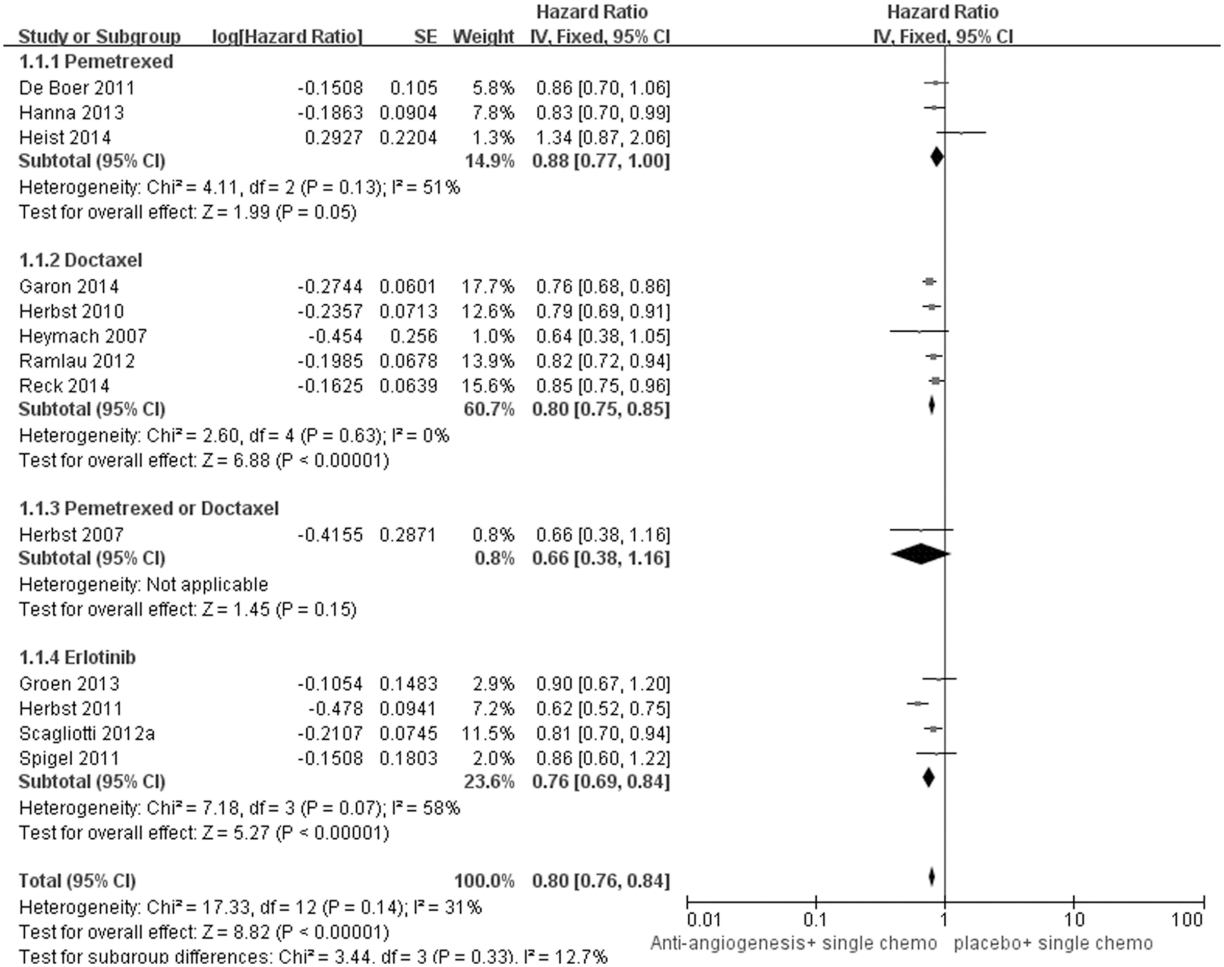
Forest plot and pooled HR & 95%CI for PFS: Antiangiogenic agents plus single agent chemotherapy versus standard second-line chemotherapy.

**Fig 4:**
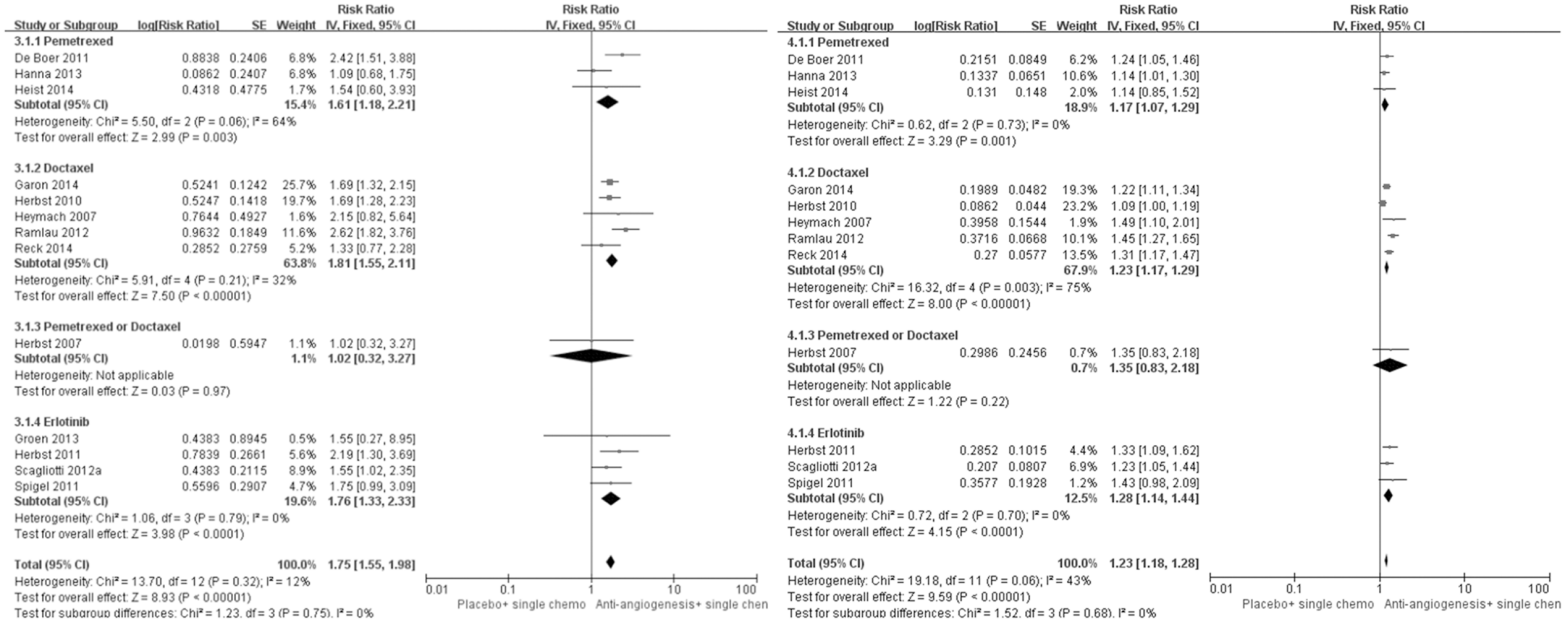
Forest plot and pooled RR & 95%CI for ORR (left) and DCR (right): Antiangiogenic agents plus single agent chemotherapy versus standard second-line chemotherapy.

All of the included studies were included for PFS and ORR analysis. However, the pooled result of DCR was based on 12 studies as one study [23] did not report the relevant data. All population analysis showed a favorable trend for the addition of angiogenesis inhibitors to the present standard second-line chemotherapy. Fig 3 indicate that the risk of disease progression was decreased by 20% compared to the chemotherapy alone, with significant pooled result (HR for PFS was 0.80, 95%CI: 0.76–0.84, p<0.00001). Meanwhile, as shown in Fig 4, this combination strategy significantly improved the DCR (Pooled RR was 1.23, 95%CI 1.18–1.28, p<0.00001) and ORR (Pooled RR was 1.75, 1.55–1.98, p<0.00001). (Table 2)

However, subgroup analysis showed that combination with angiogenesis inhibitor failed to bring additional efficacy to pemetrexed (Pooled HR for PFS 0.91, 95%CI: 0.74–1.11, p = 0.36). (Table 4)

**Table 4:** Summary of the subgroup results: Pooled HR & 95%CI for PFS and the corresponding details.

	No. of articles	Pooled HR with 95%CI	P-value	Heterogeneity (I^2^)	Analysis model
**AT** *	9	0.83 (0.78–0.89)	<0.00001	0%	Fixed
**AA** ^&^	4	0.74 (0.65–0.84)	<0.00001	51%	Random
**Pemetrexed**	3	0.91 (0.74–1.11)	0.36	51%	Random
**Doctaxel**	5	0.80 (0.75–0.85)	<0.00001	0%	Fixed
**Non-Doctaxel**	7	0.83 (0.72–0.94)	0.005	58%	Random
**EGFR-TKI**	4	0.77 (0.65–0.92)	0.003	58%	Random
**Chemotherapy**	9	0.81 (0.77–0.87)	<0.00001	10%	Fixed
**Double TKI** ^¶^	3	0.83 (0.74–0.94)	0.003	0%	Fixed

* AT for antiangiogenic-TKI;

^&^ AA refers to antiangiogenic antibody.

^¶^ Double TKI means antiangiogenic-TKI plus EGFR-TKI.

### Risk of bias and publication bias

For most studies included in this meta-analyses, low risk of bias existed for all key domains, including sequence generation, allocation concealment, blinding of participants or outcome assessment, incomplete outcome data, selective outcome reporting and other sources of bias. No high risk of bias was detected among the thirteen RCTs as shown in S1 Fig.

**Fig 5:**
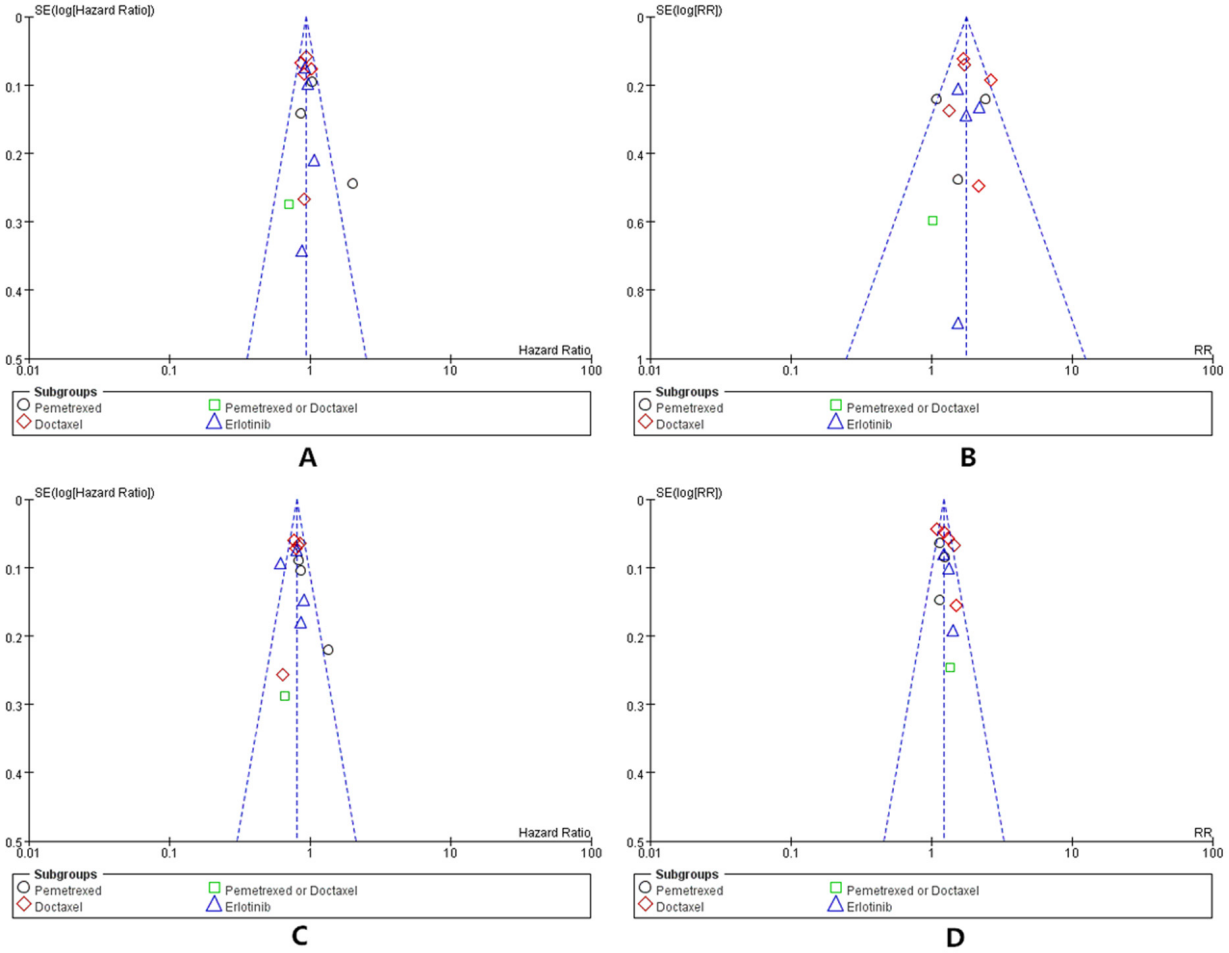
Qualitative analysis of publication bias: Funnel plot of included studies for all outcome. (A) OS, (B) ORR, (C) PFS and (D) DCR.

As shown in Fig 5, statistical analysis showed that certain publication bias actually existed during OS analysis. However, no significant publication bias was observed for other outcomes, including PFS, ORR and DCR.

## Discussion

To our knowledge, this is the first meta-analysis to assess the role of antiangiogenesis combined with chemotherapy for the NSCLC patients in second-line setting. Data from our meta-analysis indicated that the addition of antiangiogenic agents to standard treatments could provide extra benefit for advanced NSCLC patients in terms of OS, PFS, ORR and DCR in the whole population. Further subgroup analysis implied that the patients with non-squamous NSCLC might be the potential target population, and docetaxel might be the best option for the combination treatment strategy.

To date, the clinical outcome of NSCLC patients who failed from first-line treatment remains poor. Effective salvage therapies for this population are urgently needed. Considering the biological rationale for targeting angiogenesis, the combination of antiangiogenic therapy and standard treatment could be a reasonable option. However, outcomes of clinical trials evaluating this combination strategy were inconsistent.

The benefit of this strategy has been questioned by some oncologists. According to our study, the combination of antiangiogenic treatment with standard therapy could increase anti-tumor efficacy, and improve overall survival in NSCLC patients versus standard therapy alone. Thus there is still a clinical rationale for targeting angiogenesis in patients with advanced NSCLC progressed after first-line treatment.

Although improved overall survival was noted in the whole population receiving combination therapy, the subgroup assessment suggested that the OS improvement occurred only in patients treated with docetaxel plus antiangiogenic agents. The addition of antiangiogenic compounds to pemetrexed or erlotinib failed to show OS advantage. This finding was consistent with previous studies in the first-line setting [8, 27]. Preclinical researches implicated that pro-angiogenic bone marrow derived circulating endothelial progenitor (CEP) cells contributed to drug resistance and re-growth of tumor cells during the chemotherapy free break, and reduced the effectiveness of chemotherapy [28]. Furthermore, antiangiogenic drugs could block acute mobilization of CEP induced by chemotherapy, and increase anti-tumor efficacy [29]. However, different chemotherapeutic drugs have variable abilities in inducing CEP mobilization. Taxanes (paclitaxel and docetaxel) could cause acute CEP elevations within 24 hours of a single bolus injection, whereas other agents (gemcitabine, cisplatin, doxorubicin, CPT-11, and cyclophosphamide) failed to induce rapid mobilization of CEP [30]. These findings may explain the favorable anti-tumor effect of taxanes when combined with antiangiogenic drugs.

Toxicities are considered a vitally important outcomes for cancer treatment, especially in second-line setting. According to the included RCTs, combination regimen was associated with more severe side effects than standard second-line treatment alone but generally mild or moderate in severity and mostly manageable. However, given the heterogeneity of toxicity profile among various antiangiogenic and cytotoxic agents, we only conducted the analysis of toxicity-related death between the combination group and the control group. The pooled result indicated that the addition of antiangiogenic agents to standard second-line regimens slightly increased the risk of death caused by treatment toxicities (pooled RR was 1.22, 95%CI: 1.03–1.43, p = 0.02) as shown in S2 Fig. Some antiangiogenic agents, such as vandetanib, did not produce substantial additional toxicity versus chemotherapy alone [16]. However, great caution should be paid to the toxicities during treatment, especially those caused by antiangiogenic drugs, such as hypertension, bleeding, perforation and albuminuria. The selection of appropriate patients who may gain the greatest benefit from this combination approach becomes the major bottleneck of the current research.

Due to the highly heterogeneous nature of NSCLC, it is possible that specific subgroups of NSCLC patients are more likely to benefit from antiangiogenic agents. Previous studies suggested that NSCLC tumor histology influences the response to both chemotherapy and targeted therapy [31–32]. Our study also found that patients with non-squamous NSCLC had significant longer OS when treated with combination therapy versus chemotherapy alone, whereas patients with squamous cell carcinoma failed to gain OS benefit from additional antiangiogenic therapy. These results suggested that patients with non-squamous NSCLC might be the targeted sub-population for antiangiogenic treatments. However, the underlying biological reason is yet unclear. Clinical experience with EGFR inhibitors indicates that antiangiogenic drugs will be most effective in patients with specific molecular variants. Several biomarkers have been evaluated as predictive factors for antiangiogenic therapy, including VEGF-A, VEGFR, placental growth factor (PLGF), neuropilin-1 (NRP-1) and so on [33–35]. Unfortunately, to date, no validated biomarker has been identified for any angiogenesis inhibitor. Owing to lack of available biomarkers to identify the precise targeted population, selecting patients by tumor histology would be an acceptable strategy.

Our meta-analysis has several limitations. Firstly, this study may suffer from clinical heterogeneity due to the involvement of various standard treatment regimens and antiangiogenic agents. Secondly, our study is based on data abstracted from publications instead of individual patient data, which could offer more useful information. Finally, for certain subgroup analysis, publication bias existed due to unclear reasons. Publication status may be one of the contributing factors as ongoing studies were ineligible for inclusion.

In conclusion, our study revealed that adding antiangiogenic agents to standard treatments could provide clinical benefits to NSCLC patient who failed their first-line therapy. Furthermore, proper selection of the standard treatment regimens and patients population by tumor histology is substantial for future studies and clinical application of antiangiogenic therapy.

## Supporting Information

S1 Checklist PRISMA Checklist. (PDF)

S1 Fig Risk of bias graph (above) and Risk of bias summary (bottom): review authors' judgements about each risk of bias item presented as percentages across all included studies. (TIF)

S2 Fig Forest plot and pooled RR & 95%CI for toxicity-related death: Antiangiogenic agents plus single agent chemotherapy versus standard second-line chemotherapy. (TIF)
