# Supplementary material for: The Efficacy of Combining Antiangiogenic Agents with Chemotherapy for Patients with Advanced Non-Small Cell Lung Cancer Who Failed First-Line Chemotherapy: A Systematic Review and Meta-Analysis
Source: PLoS One. 2015 Jun 2;10(6):e0127306. doi: 10.1371/journal.pone.0127306 (PMC4452723; doi:10.1371/journal.pone.0127306)
Supplement: S1 Checklist — (PDF) [file pone.0127306.s001.pdf]

PRISMA 2009 Checklist

Section/topic	#	Checklist item	Reported on page #
**TITLE**			
Title	1	The Efficacy of Combining Antiangiogenic Agents with Chemotherapy for Patients with Advanced Non-Small Cell Lung Cancer who Failed First-line Chemotherapy: A Systematic Review and Meta-analysis	1
**ABSTRACT**			
Structured summary	2	Background: The clinical outcomes in patients with NSCLC progressed after first-line treatments remain poor. The purpose of this study was to assess the advantage of antiangiogenic therapy plus standard treatment versus standard treatment alone for this patient population.<br>Methods: We conducted a rigorous search from electronic databases for eligible studies reporting antiangiogenic therapy combined with standard second-line chemotherapy versus standard second-line treatment for patient progressed after front-line treatment. Pooled risk ratio and 95% confidence intervals were calculated using proper statistical method. Predefined subgroup analyses were conducted to identify the potential proper patient.<br>Results: Thirteen phase II/III RCTs which involved a total of 8358 participants were included. Overall, there was significant improvement in OS (HR 0.93, 95%CI: 0.88-0.99, p=0.01), PFS (HR 0.80, 95%CI: 0.75-0.86, p<0.00001), ORR (RR 1.75, 95%CI: 1.55-1.98, p<0.00001) and DCR (RR 1.23, 95%CI: 1.18-1.28, p<0.00001) in group with anti-angiogenic therapy plus standard treatment versus group with standard treatment alone. Subgroup analyses showed that OS benefit were presented only in patients treated with docetaxel plus antiangiogenic agents (HR 0.92, 95%CI: 0.86-0.99, p=0.02) and patients with non-squamous NSCLC (HR for OS 0.92, 95%CI: 0.86-0.99, p=0.02).<br>Conclusions: This study revealed that the addition of antiangiogenic agents to standard treatments could provide clinical benefit to NSCLC patient who failed from first-line therapy. Furthermore, proper selection of the combined standard cytotoxic agent and patient population by tumor histology is substantial for future studies and clinical application of antiangiogenic therapy.<br>Keywords: NSCLC, Angiogenesis, Meta-analysis	2
**INTRODUCTION**			
Rationale	3	The roll of antiangiogenic therapy has been well recognized in first-line treatment for NSCLC patients. Two meta-analyses indicate significant improvement of ORR, PFS, and OS for the combination of antiangiogenic agent (bevacizumab) and chemotherapy compared with chemotherapy alone. Several clinical guidelines also recommend the addition of bevacizumab to standard treatment in the first-line setting]. However, the advantage of adding antiangiogenic agent to the standard treatment in patients failed from first-line therapy is still confusing.	3
Objectives	4	To compare the efficacy of angiogenesis inhibitors plus santard treatment versus santard treatment alone for patients with advanced NSCLC progressed after first-line treatment.	4
**METHODS**			
Protocol and registration	5	The data collection and assessment of methodological quality followed the QUORUM and the Cochrane Collaboration guidelines (http://www.cochrane.de).Meta-analyses was conducted according to the Preferred Reporting Items for Systematic Reviews and Meta-Analyses (PRISMA) statement. The PRISMA checklist was showed in S1 File.	4,5

PRISMA 2009 Checklist

Section/topic	#	Checklist item	Reported on page #
Eligibility criteria	6	Studies that met the following criteria were included: (1) Adult (⩾18 years) patients with histologically or cytologically confirmed stage IIIB/IV NSCLC (all histologies); (2) Phase II or III RCTs that evaluate the efficacy of angiogenesis inhibitors plus present standard single agent chemothrapy (pemetrexed, doctaxel or erlotinib) as salvage cure for patient progressed after first-line treatent; (3) The control group must be the corresponding cytotoxic agent; (4) At least one endpoints (PFS, OS, ORR and DCR) was reported. Trials were excluded if they fail to meet the including criteria. In cases of duplicate trials, the most complete reports were included.	4
Information sources	7	All relevant articles were retrieved by searching PubMed, Embase and the Central Registry of Controlled Trials of the Cochrane Library.	4
Search	8	In October 2014, all relevant articles were retrieved by searching through PubMed, Embase and the Central Registry of Controlled Trials of the Cochrane Library, as well as the ASCO and ESMO databases. Search strategety were the combination of “non-small-cell lung cancer” with any of the following: "angiogenesis inhibitors" or “sorafenib”, "sunitinib”, ”bevacizumab”, “vandetanib”, “aflibercept”, “nintedanib”, “pazopanib”, "ramcirumab" or “axitinib”. Recent reviews and references of the included studies and were checked manually as a supplement. No language restriction was applied.	4
Study selection	9	The data collection and assessment of methodological quality followed the QUORUM and the Cochrane Collaboration guidelines (http: //www. cochrane.de). Researcher evaluated the quality of each eligible study according to the JADAD score.	5
Data collection process	10	Two authors carried out the search independently. The data collection and assessment of methodological quality followed the QUORUM and the Cochrane Collaboration guidelines (http://www.cochrane.de). Discrepancies were discussed by the third investigators to reach consensus. We tried to obtain additional unpublished data by contacting the primary authors to obtain unpublished data.	5
Data items	11	Baseline clinical characteristics, total number of enrolled participants, the risk ratio (RR) and 95% confidence intervals (CI) for objective response rates (ORR) and disease control rates (DCR), median value, hazard ratio (HR) of overall survival (OS) and progression-free survival (PFS), were extracted.	5
Risk of bias in individual studies	12	An extensive search strategy was made to minimize the potential for publication bias. Graphical funnel plots were generated to visually assess a publication bias. The statistical methods to detect funnel plot asymmetry were the rank correlation test of Begg and Mazumdar and the regression asymmetry test of Egger.	5
Summary measures	13	Pooled hazard ratios (HRs) for survival outcomes (PFS and OS) and pooled risk ratio (RRs) for dichotomous data (ORR, DCR) with 95% CI were calculated using the proper algorithm.	5
Synthesis of results	14	Pooled hazard ratios (HRs) for survival outcomes (PFS and OS) and pooled risk ratio (RRs) for dichotomous data (ORR, DCR) with 95% CI were calculated using the proper algorithm. Heterogeneity across studies was assessed with a forest plot and the inconsistency statistic (I^2). A random-effects model was employed in case of the existence of potential heterogeneity ($I^2 \geqslant 50\%$); otherwise, the fixed-effect model was applied. All calculations were performed using Review Manager (version 5.2 for Windows; the Cochrane Collaboration, Oxford, UK). Graphical funnel plots were generated to visually inspect for publication bias. P<0.05 was considered statistically significant for all analysis.	5,6

Risk of bias across studies	15	All calculations and the assessement of risk of bias were performed using Review Manager (version 5.2 for Windows; the Cochrane Collaboration, Oxford, UK).	6
Additional analyses	16	Graphical funnel plots were generated to visually inspect for publication bias. P<0.05 was considered statistically significant for all analysis.	6
**RESULTS**			
Study selection	17	Twenty potentially eligible trials were rigorously identified by full-text review, 7 of which were excluded for reasons listed in Figure 1. Finally, 13 studies with 8358 patients met the inclusion criteria were included for the analysis.	6
Study characteristics	18	As respect to the type of standard second-line cytototic agents, the number of studies involved pemetrexed, docetaxel and EGFR-TKI were separately 3, 5, 4. Another onewas designed to illustrate the efficacy of the addition of bevacizumab to docetaxel or pemetrexed. Four studies reported the result of combination of antiangiogenic MAs, the remaining nine studies were about single agent chemotherapy combined with VEGF-TKI or placebo. In further subgroup exploration, the efficacy of "double TKIs"model, which implying antiangiogenic TKI combined with EGFR-TKI, was evaluated based on 3 RCTs. As regards histological type, nine studies provided relevant subgroup information. Detailed information of included studies was listed in Table 1. The specific number of included study may vary according to the corresponding outcomes.	6
Risk of bias within studies	19	For most studies included in this meta-analyses, low risk of bias existed for all key domains, including sequence generation, allocation concealment, blinding of participants or outcome assessment, incomplete outcome data, selective outcome reporting and other sources of bias. No high risk of bias was detected among the thirteen RCTs as shown in S2 Fig.	8
Results of individual studies	20	Table 3 and Table4 summarized the detail of subgroup results for OS and PFS.	7,8
Synthesis of results	21	In general, for patients progressed after front-line chemotherapy, the addition of angiogenesis inhibitors was associated with significant superior survival improvement compared with standard second-line single agent chemotherapy, reducing 5% of the risk of death (HR for OS 0.93, 95%CI: 0.88-0.99, p=0.01). (Table 2) As listed in Table 3, the pooled result indicated that patients with non-squamous cancer benefit most from the combination strategy (Pooled HR for OS 0.92, 95%CI: 0.86-0.99, p=0.02). In addition, the pooled result was in favor of the combination of docetaxel with angiogenesis, which significant improved the overall survival for patient underwent progression after first-line chemotherapy (pooled HR for OS was 0.92, 95%CI: 0.86-0.99, p=0.02). Angiogenensis inhibitor combined with pemetrexed or erlotinib provided slightly improved OS, however, the difference was not significant compared with chemotherapy alone. With respect to angiogenesis inhibitors, monoclonal antibody was only numerically superior to VEGF-TKI in decreasing the risk of death (Pooled HR were separately 0.93, 95%CI: 0.85-1.01, p=0.08 and 0.95, 95%CI: 0.89-1.02, p=0.16). Meanwhile, the distinguish combination of anti-angiogenetic-TKI and EGFR-TKI slightly decreased the risk of death, however, the difference was not statistically significant (Pooled HR 0.94, 95%CI: 0.82-1.07, p=0.34). All population analysis showed a favorable trend for the addition of angiogenesis inhibitors to the present standard second-line chemotherapy. Figure 3 indicated that the risk of disease progression was decreased 20% compared with chemotherapy alone, with significant pooled result (HR for PFS was 0.80, 95%CI: 0.75-0.86, p<0.00001). Meanwhile, as shown in Figure 4, this combination strategy significantly improved the DCR (Pooled RR was 1.23, 95%CI 1.18-1.28, p<0.00001) and ORR (Pooled RR was 1.75, 1.55-1.98, p<0.00001). (Table 2) However, subgroup analyses showed that combination with angiogenesis inhibitor failed to bring about additional efficacy to pemetrexed (Pooled HR for PFS 0.91, 95%CI: 0.74-1.11, p=0.36). (Table 4)	7,8

PRISMA 2009 Checklist

Risk of bias across studies	22	The publication bias was detected by Begg' s test and Egger' s test. No significant publication bias was observed for all out-comes (p > 0.05).Figure 5	8
Additional analysis	23	The publication bias was detected by Begg's test and Egger's test. As shown in Fig.5, no significant publication bias was observed for all out-comes (p > 0.05).	8
**DISCUSSION**			
Summary of evidence	24	Data from our meta-analysis indicated that the addition of antiangiogenic agents to standard treatments could provide benefit for advanced NSCLC patients in terms of OS, PFS, ORR and DCR in the whole population. Further subgroup analyses implied that the patients with non-squamous NSCLC might be the potential target population, and docetaxel might be the better choice for the combination treatment strategy.	8,9
Limitations	25	Our meta-analysis has several limitations. Firstly, this study may suffer from clinical heterogeneity due to involving various standard treatment regimens and anti-angiogenic agents. Secondly, our study is based on data abstracted from publications instead of individual patient data, which could offer more useful information especially for subgroup assessment. Finally, for certain subgroup analyses some publications are excluded due to lack of the specific information required.	10
Conclusions	26	In conclusion, our study revealed that the addition of antiangiogenic agents to standard treatments could provide clinical benefit to NSCLC patient who failed from first-line therapy. Furthermore, proper selection of the standard treatment regimens and patients population by tumor histology is substantial for future studies and clinical application of antiangiogenic therapy.	10
**FUNDING**			
Funding	27	1. National High Technology Research and Development Program of China (Grant No. 2012AA02A502)<br>2. Innovative drug R&D center based on real-time high-throughput cell-based screening platform and large capacity compound library (Grant No. 2013ZX09401003-002)<br>3. National Natural Science Funds of China (Grant No. 81372502)<br>4. Wu Jieping Medical Foundation Project (Grant No. 320.6750.131)<br>All the funders had no role in study design, data collection and analysis, decision to publish, or preparation of the manuscript.	11

From: Moher D, Liberati A, Tetzlaff J, Altman DG, The PRISMA Group (2009). Preferred Reporting Items for Systematic Reviews and Meta-Analyses: The PRISMA Statement. PLoS Med 6(6): e1000097. doi:10.1371/journal.pmed1000097
