# Supplementary figures and images for: The Efficacy of Combining Antiangiogenic Agents with Chemotherapy for Patients with Advanced Non-Small Cell Lung Cancer Who Failed First-Line Chemotherapy: A Systematic Review and Meta-Analysis
Source: PLoS One. 2015 Jun 2;10(6):e0127306. doi: 10.1371/journal.pone.0127306 (PMC4452723; doi:10.1371/journal.pone.0127306)

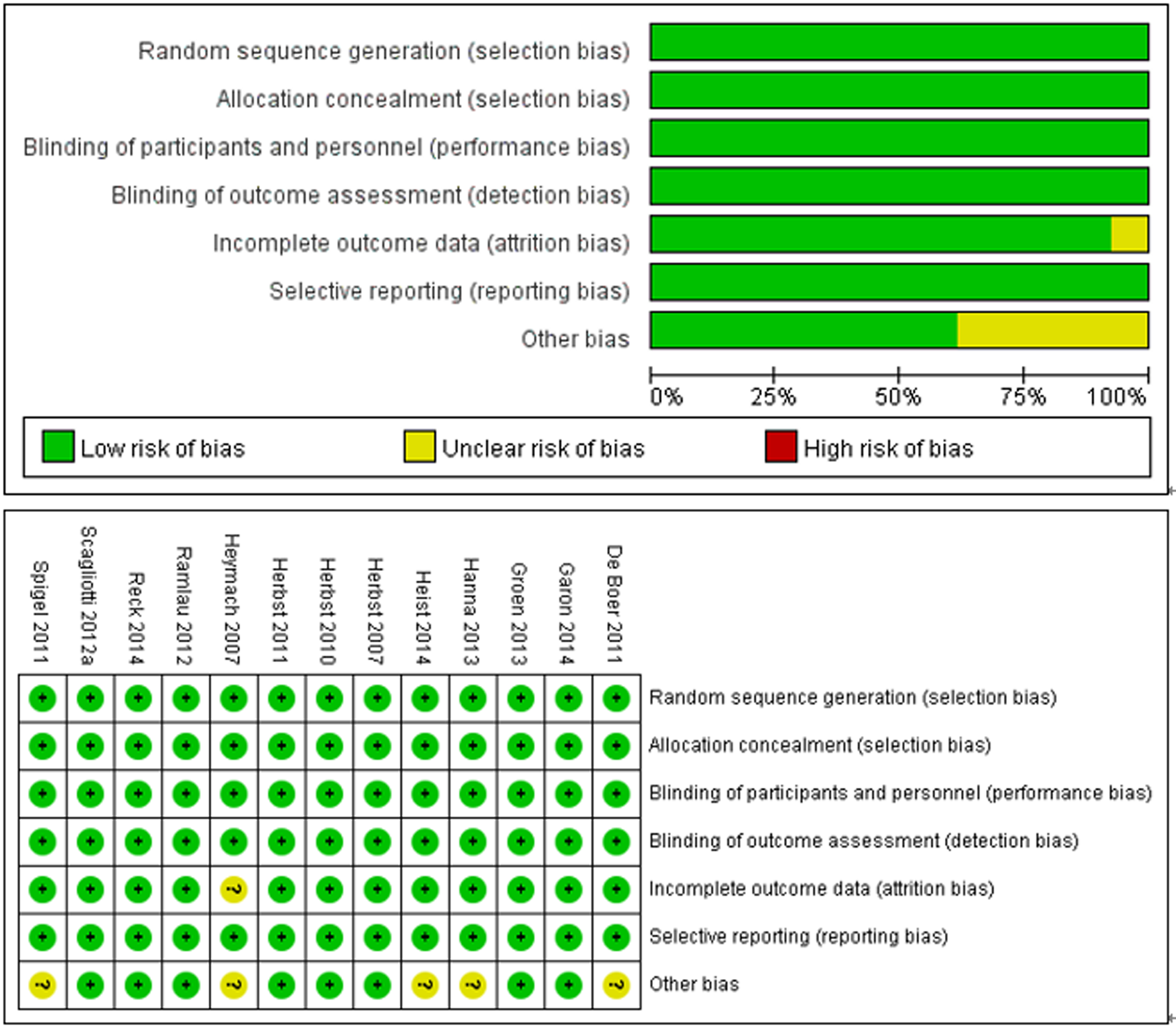

Supplement: S1 Fig — (TIF) [file pone.0127306.s002.tif]
